# Cross-bridge thermodynamics in pulmonary arterial hypertensive right-ventricular failure

**DOI:** 10.1152/japplphysiol.00014.2022

**Published:** 2022-04-28

**Authors:** Toan Pham, Kenneth Tran, Andrew J. Taberner, Denis S. Loiselle, June-Chiew Han

**Affiliations:** ^1^Auckland Bioengineering Institute, The University of Auckland, Auckland, New Zealand; ^2^Department of Engineering Science, The University of Auckland, Auckland, New Zealand; ^3^Department of Physiology, The University of Auckland, Auckland, New Zealand

**Keywords:** cardiac trabeculae, cross-bridge thermodynamics, dynamic stiffness, mechano-energetics, right-ventricular failure

## Abstract

Right-ventricular (RV) failure is an event consequent to pathological RV hypertrophy commonly resulting from pulmonary arterial hypertension. This pathology is well characterized by RV diastolic dysfunction, impaired ejection, and reduced mechanical efficiency. However, whether the dynamic stiffness and cross-bridge thermodynamics in the failing RV muscles are compromised remains uncertain. Pulmonary arterial hypertension was induced in the rat by injection of monocrotaline, and RV trabeculae were isolated from RV failing rats. Cross-bridge mechano-energetics were characterized by subjecting the trabeculae to two interventions: *1*) force-length work-loop contractions over a range of afterloads while measuring heat output, followed by careful partitioning of heat components into activation heat and cross-bridge heat to separately assess mechanical efficiency and cross-bridge efficiency, and *2*) sinusoidal-perturbation of muscle length while trabeculae were actively contracting to interrogate cross-bridge dynamic stiffness. We found that reduced mechanical efficiency is correlated with increased passive stress, reduced shortening, and elevated activation heat. In contrast, the thermodynamics, specifically the efficiency of, and the stiffness characteristics of, cross bridges did not differ between the control and failing trabeculae and were not correlated with elevated passive stress or reduced shortening. We thus conclude that, despite diastolic dysfunction and mechanical inefficiency, cross-bridge stiffness and thermodynamics are unaffected in RV failure following pulmonary arterial hypertension.

**NEW & NOTEWORTHY** This study characterizes cross-bridge mechano-energetics and dynamic stiffness of right-ventricular trabeculae isolated from a rat model of pulmonary hypertensive right-ventricular failure. Failing trabeculae showed increased passive force but normal active force. Their lower mechanical efficiency is found to be driven by an increase in the energy expenditure arising from contractile activation. This does not reflect a change in their cross-bridge stiffness and efficiency.

## Graphical abstract

**Figure:**
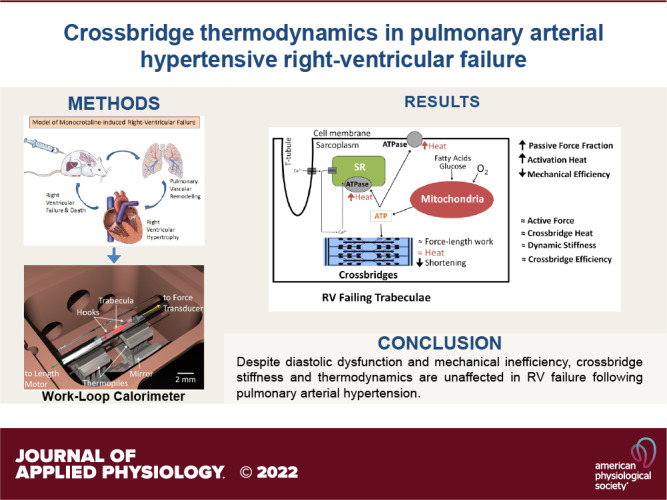

## INTRODUCTION

The right ventricle (RV) is increasingly gaining research attention given the prognostic significance of RV failure. In pulmonary arterial hypertension, a pathological rise in pulmonary vascular resistance and pulmonary vasoconstriction overload the RV, leading to RV hypertrophy and culminating in RV failure (1, 2). During the disease progression, diastolic dysfunction is observed in vivo clinically (3), as well as experimentally in ex vivo heart preparations (4), in vitro muscle tissues (5, 6), and single myocytes (3, 7). Diastolic dysfunction is manifested by an increase of RV diastolic pressure (4, 8) or passive stress (3, 5–7) and is associated with a decrease of RV ejection fraction or shortening fraction (5, 9–12) and slower contractile activity (5, 13). An increase in the severity of RV hypertrophy has been reported to be associated with a decrease in RV ejection fraction (14) and a decrease in mechanical efficiency (15). These observations suggest a positive correlation between RV ejection fraction and mechanical efficiency (16).

The reduction of muscle mechanical efficiency in both human patients (16) and rats (15) with RV failure is attributed to the increased energy expenditure indexed as myocardial oxygen consumption since the mechanical work output is shown to be largely unaffected (15, 16). Myocardial energy expenditure during active contraction funds cellular processes for the cycling of Ca^2+^ and the cycling of cross bridges. The increased energy expenditure in RV failure is revealed to arise from the increased energy cost associated with cycling of Ca^2+^ required for the activation of contraction (5). What remains unquantified is the energy cost associated with cross-bridge cycling, specifically the extent to which the thermodynamic efficiency of cross bridges per se contributes to the diminution of mechanical efficiency, over a wide range of workloads.

Although diastolic dysfunction has been observed, studies using cardiac muscles (5, 15) and cardiomyocytes (17) isolated from RV failure models have, nevertheless, found the active stress production to be equal in magnitude to that developed by the control samples. We, therefore, hypothesized that if active force production is preserved in RV failure, then the number of cycling cross bridges, the energy cost for cross-bridge cycling, and, hence the thermodynamics efficiency of cross bridges will be preserved, too. To test this hypothesis, we characterized muscle dynamic stiffness and cross-bridge efficiency as distinct from muscle mechanical efficiency, by measuring muscle stress production and energy expenditure in RV trabeculae isolated from failing hearts with monocrotaline (MCT)-induced pulmonary arterial hypertension. This investigation was rendered possible by our recent study that establishes a protocol for decomposing the energy expenditure into its activation and cross-bridge components (18). The protocol allows quantification of cross-bridge heat output and, thereby, cross-bridge efficiency. To that end, we assessed the correlations among several measured parameters including the degree of RV hypertrophy, developed passive and active stresses, shortening fraction, work output, mechanical efficiency, cross-bridge heat output, and, ultimately, cross-bridge efficiency.

## METHODS

### Animal Preparation

Protocols for animal preparation were approved by the Animal Ethics Committee of The University of Auckland (No. R1403). Male Wistar rats (300–325 g) were randomly divided into two groups. Failing (“FAIL”) group received a single injection of monocrotaline (MCT) of 60 mg·kg^−1^, whereas Control (“CON”) group received an equivalent volume of saline. We (19) have previously reported cardiac hemodynamic changes in vivo commencing at *week 4* postinjection in MCT-treated rats, including significant changes in heart rate and decreasing systolic, diastolic, and mean arterial blood pressures. At *week 6* postinjection, the MCT-treated rats were expected to transition to RV failure, following our previous confirmation from in vivo telemetric measurements to show bradycardia, higher RV systolic pressure, and slower contractile kinetics in terms of the rates of rise and fall of RV developed pressure (19). Thus, the present study euthanized rats for experiments at *week 6* postinjection. Signs of heart failure also included consecutive days of weight loss (>15%), dyspnea, lethargy, and piloerection.

### Trabecula Preparation

On the day of an experiment, a rat was anesthetized with isoflurane and weighed before receiving an injection of heparin (1,000 IU·kg^−1^). Following a cervical dislocation, the heart was excised, plunged into cold Tyrode solution, and immediately Langendorff perfused with oxygenated Tyrode solution at room temperature. The solution, with pH adjusted to 7.4 using Tris, contained (in mmol·L^−1^) 130 NaCl, 6 KCl, 1 MgCl_2_, 0.3 CaCl_2_, 0.5 NaH_2_PO_4_, 10 HEPES, 10 glucose, and 20 2,3-butanedione monoxime. Trabeculae were excised from the endocardial surface of the RV and mounted in a work-loop calorimeter (20, 21).

In the calorimeter, the ends of the trabecula were held by platinum hooks for length control (upstream) and force measurement (downstream). The trabecula was superfused with the same oxygenated Tyrode solution but containing 1.5 mmol·L^−1^ CaCl_2_ and without 2,3-butanedione monoxime. Superfusate flow rate was electronically controlled at 0.55 μL·s^−1^ for optimal thermal sensitivity of the calorimeter (22) while ensuring negligible risk for the trabecula developing a hypoxic core (23). The temperatures of the superfusate upstream and downstream of the trabecula were measured by thermopile arrays embedded beneath the measurement chamber in the calorimeter. The increase in temperature downstream relative to that upstream was proportional to the rate of heat liberation by the trabecula. The trabecula was stimulated, via a pair of platinum electrodes, to contract at 3 Hz to allow a stepwise, gradual, stretch to optimal length (*L_o_*), thereby achieving the maximal active force production. The entire calorimeter system was optically isolated and thermally insulated in its enclosure, and the interior temperature was electronically maintained at 37°C (24).

### Measurement of Mechanical Efficiency

Stimulus frequency was increased to 5 Hz. The contracting trabecula was required to undergo a series of stress-length loops at a range of afterloads, as previously described (25, 26). The rate of muscle active heat output was simultaneously measured. Mechanical work output was defined by the area of the stress-length loop, which was calculated by integrating stress with respect to muscle length throughout the time course of a contractile twitch.

Mechanical efficiency of the trabecula was quantified as the ratio of mechanical work output to the suprabasal, active, energy expenditure (the sum of work and active heat). The suprabasal active heat comprised the thermal output arising from the splitting of ATP required for two cellular activities: Ca^2+^ cycling (activation heat) and cross-bridge cycling (cross-bridge heat).

### Measurement of Cross-Bridge Efficiency

The trabecula was subsequently subjected to a series of isometric contractions at various muscle lengths from *L_o_* to near 0.75 *L_o_* (where active stress was minimal). This protocol allowed quantification of activation heat, defined as the intercept of the heat-stress relation (18). Cross-bridge heat was quantified by subtracting activation heat from the heat measured under work-loop contractions. Cross-bridge efficiency was defined as the ratio of work output to the sum of work and cross-bridge heat.

### Measurement of Cross-Bridge Dynamic Stiffness

To interrogate cross-bridge dynamic stiffness, muscle length was sinusoidally perturbed by the length-motor upstream at 100 Hz with a constant amplitude of 0.001 *L_o_*, as previously described in detail (27). Dynamic stiffness was estimated from the ratio of the resulting sinusoidal change of force to change of length. Dynamic modulus was defined by the product of dynamic stiffness and the ratio of muscle length to muscle cross-sectional area. A total of 6 CON and 9 FAIL trabeculae were subjected to the measurement of cross-bridge dynamic stiffness.

### Measurement of Muscle Dimensions and Definitions

Each trabecula was assumed to resemble an ellipse in cross section and, hence, muscle force was converted to stress from the measurement of muscle diameters in two orthogonal views (top and side), where the side was viewed via a mirror located at 45° in the measurement chamber. In total, 8 CON trabeculae and 13 FAIL trabeculae were studied for the measurements of efficiency. They were isolated from 5 CON rats and 9 FAIL rats, respectively.

There were no statistically significant differences, using Student’s unpaired *t* test, in trabecula dimension between CON and FAIL trabeculae in either cross-sectional area (means ± standard deviation: 0.081 mm^2^ ± 0.022 mm^2^ and 0.079 mm^2^ ± 0.019 mm^2^, respectively) or muscle volume (means ± standard deviation: 0.278 mm^3^ ± 0.091 mm^3^ and 0.231 mm^3^ ± 0.087 mm^3^, respectively).

Force was converted to stress (kPa) by normalizing to muscle cross-sectional area. Twitch heat (kJ·m^−3^) was calculated by dividing the steady-state rate of heat production by the stimulus frequency (5 Hz) and normalizing it to muscle volume. Work output (kJ·m^−3^) was calculated by integrating stress as a function of relative muscle length over the period of the twitch.

### Correction for Thermal Artifacts

At the completion of the experiment, muscle heat output was corrected for two thermal artifacts. First was the heat produced from the cyclic movement of the upstream hook in perturbing muscle length sufficient to allow the trabecula to perform stress-length loops. This artifactual heat was quantified by oscillating the trabecula in its quiescent state with stimulation halted. The second was the heat produced from electrical stimulation, which was quantified at the completion of an experiment with the trabecula removed from the calorimeter. Both heat artifacts were no more than an average of 5% of measured maximal heat output.

### Statistical Analyses

The Student’s unpaired *t* test was used to evaluate the differences between the means of the two groups. Values are means ± standard errors unless stated otherwise. A significant difference was declared at *P* < 0.05. Data were plotted against one another and were fitted using linear regressions, where correlations between two variables were assessed using Spearman’s correlation coefficient, and *P* < 0.05 was considered to demonstrate a significant correlation. We used Spearman’s correlation to test whether the two variables being compared are monotonically correlated even if their relationship is not linear. This is in contrast with Pearson correlation where the underlying assumption is that the relationship between the two variables is linear.

## RESULTS

### Morphometrics of the Failing Rat

MCT-induced RV failing (“FAIL”) rats showed morphometric characteristics that were consistent with hypertensive RV failure: lower body mass, higher lung mass, greater heart mass, including RV mass and wall thickness (Table 1).

**Table 1.** Morphometric characteristics of control and failing rats at euthanasia

	CON Rats (*n* = 5)	FAIL Rats (*n* = 9)
Body mass, g	471 ± 21	410 ± 13*
Tibial length, mm	43.0 ± 0.7	42.4 ± 0.5
Lung		
Mass, g	1.65 ± 0.07	2.36 ± 0.17*
Mass/body mass, %	0.35 ± 0.02	0.58 ± 0.04*
Mass/tibial length g·m^−1^	38.4 ± 1.7	55.4 ± 3.5*
Heart		
Mass, g	1.34 ± 0.05	1.59 ± 0.03*
Mass/body mass, %	0.28 ± 0.01	0.39 ± 0.01*
Mass/tibial length, g·m^−1^	31.1 ± 1.3	37.4 ± 0.8*
Right ventricle		
Mass, g	0.26 ± 0.01	0.55 ± 0.02*
Mass/heart mass	0.19 ± 0.01	0.35 ± 0.01*
Mass/tibial length g·m^−1^	6.0 ± 0.3	13.1 ± 0.4*
Wall thickness, mm	1.50 ± 0.04	1.91 ± 0.07*
Wall thickness/heart mass, mm·g^−1^	1.13 ± 0.06	1.21 ± 0.05
Wall thickness/tibial length, %	3.50 ± 0.12	4.51 ± 0.17*

Values are means ± standard errors. *P < 0.05. CON, control; FAIL, failing.

### Energetic Performance

**Figure 1.**
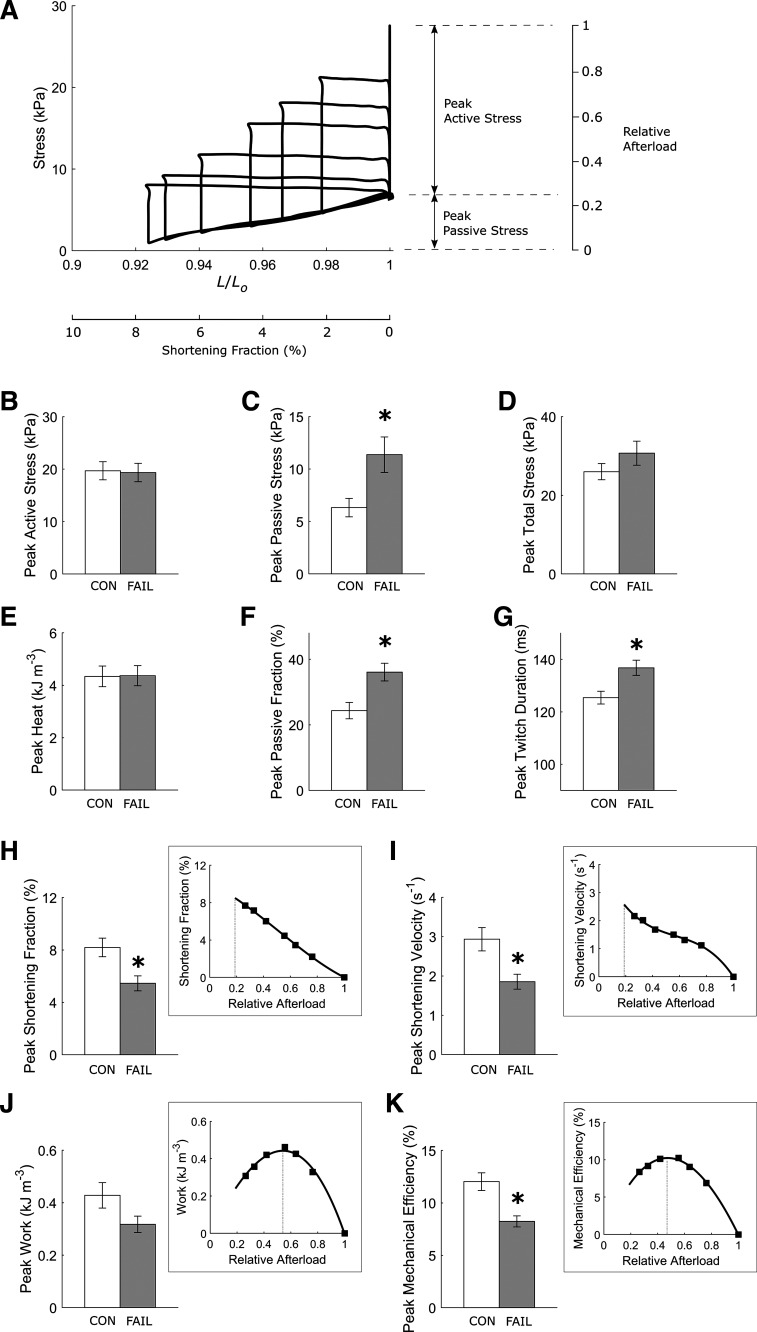
Energetic parameters are extracted from a series of afterloaded work-loop contractions. Steady-state stress-length work-loops at various afterloads from a single representative trabecula (*A*). Average values of parameters are shown (*B–K*), where *statistically significant differences between the CON (*n* = 8) and the FAIL (*n* = 13) trabeculae. Each inset in *H–K* illustrates the interpolation of the parameter of interest at the passive stress (*H* and *I*), or at the stress that maximizes work (*J*) or that maximizes mechanical efficiency (*K*), as indicated by the thin broken vertical lines. Values are presented as means ± SE. CON, control; FAIL, failing.

Isolated RV trabeculae were required to perform work-loop contractions at various afterloads (Fig. 1*A*). Peak active stress was not different between the FAIL group and the CON group (Fig. 1*B*). The heat output from developing peak active stress was also not different between groups (Fig. 1*E*). However, peak passive stress (Fig. 1*C*) and, hence peak passive stress fraction (the ratio of passive stress to total stress; Fig. 1*F*) were greater in the FAIL group. The FAIL group had slower kinetics of twitch stress, as evidenced by their prolonged twitch duration (quantified at 95% peak active stress; Fig. 1*G*) and lower peak velocity of shortening (Fig. 1*I*). The peak extent of shortening as a fraction of *L_o_* was lower in the FAIL group (Fig. 1*H*), but peak work output was not significantly different (*P* = 0.080) from the CON group (Fig. 1*J*). Peak mechanical efficiency was significantly lower in the FAIL group (Fig. 1*K*).

### Correlations of Mechano-Energetic Parameters with Morphometric Parameters

**Figure 2.**
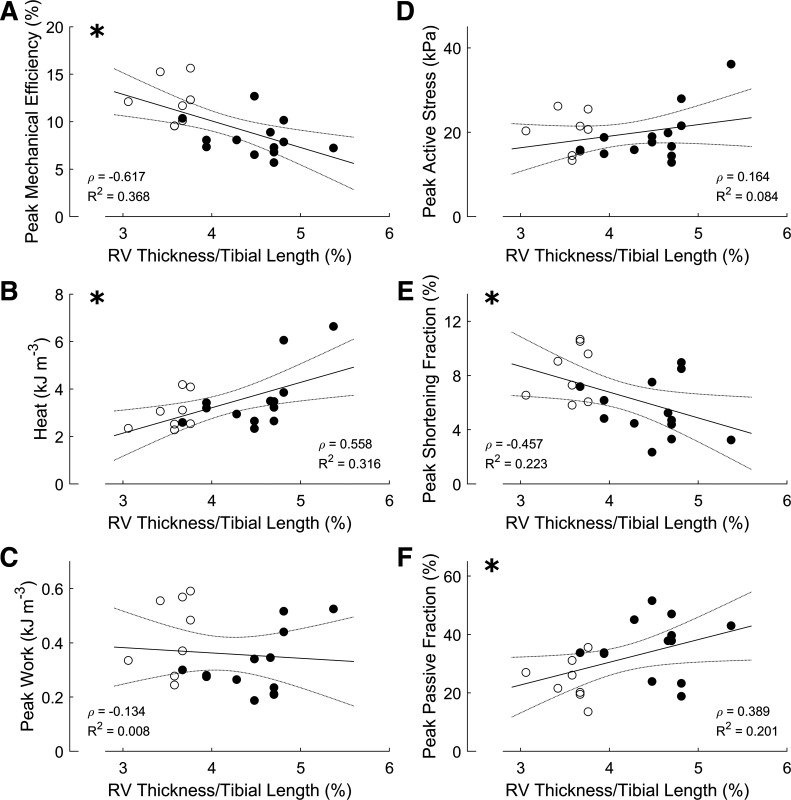
Correlations among mechano-energetic and morphometric parameters. In all panels (*A–F*), each data point was obtained from a single trabecula. Data were fitted using linear regression, displayed as a solid line, and associated with 95% confidence intervals, displayed by the concave lines. Open symbols indicate CON group; filled symbols indicate FAIL group. Statistical significance of the correlation is indicated by the presence of an * at the *top left* corner. Spearman’s correlation coefficient (ρ) and *R*^2^ values are shown in each panel. CON, control; FAIL, failing; RV, right ventricle.

Peak mechanical efficiency was negatively correlated with RV thickness normalized to tibial length (Fig. 2*A*), whereas the heat output at the peak efficiency was positively correlated with normalized RV thickness (Fig. 2*B*). Neither peak work output (Fig. 2*C*) nor peak active stress (Fig. 2*D*) was correlated with normalized RV thickness. The peak shortening fraction varied inversely with normalized RV thickness (Fig. 2*E*), whereas the peak passive fraction showed positive dependence (Fig. 2*F*). The use of multiple trabeculae from the same heart has the merit of allowing testing of data consistency or variability. The variation of functional performance of trabeculae outweighs the variation of heart morphology. For example, in Fig. 2*A*, the two trabeculae from the same heart (RV thickness/tibial length of ∼4%) had very similar peak mechanical efficiency, whereas those from the other heart (RV thickness/tibial length of ∼4.5%) had a larger variation of peak mechanical efficiency.

### Correlations among Mechano-Energetic Parameters

**Figure 3.**
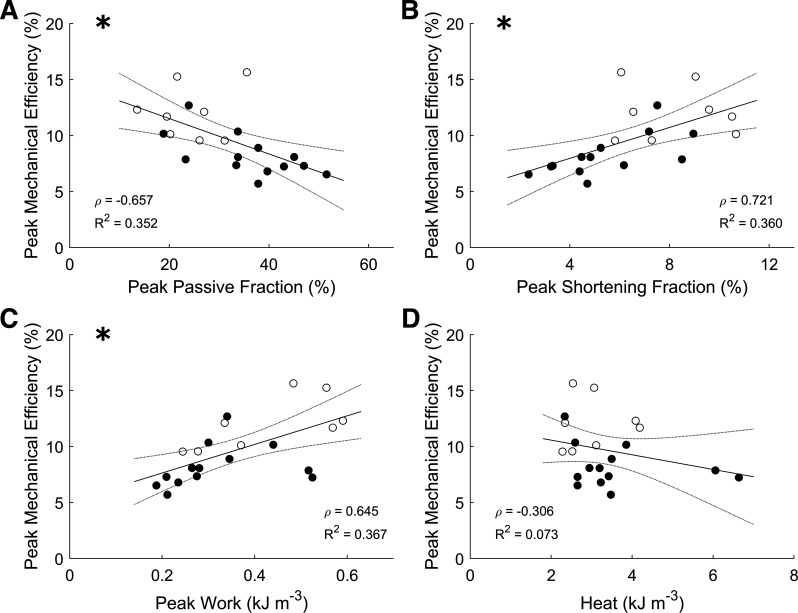
Correlations among peak mechanical efficiency and mechano-energetic parameters (*A–D*). The plotting convention is the same as in Fig. 2, where open symbols indicate CON group; filled symbols indicate FAIL group, and statistical significance of the correlation is indicated by the presence of an * at the *top left* corner. Spearman’s correlation coefficient (ρ) and *R*^2^ values are shown in each panel. CON, control; FAIL, failing.

Peak mechanical efficiency was negatively correlated with peak passive fraction (Fig. 3*A*), but positively correlated with both peak shortening fraction (Fig. 3*B*) and peak work (Fig. 3*C*). Peak mechanical efficiency was uncorrelated (*P* = 0.176) with the heat output at the point of peak mechanical efficiency (Fig. 3*D*).

**Figure 4.**
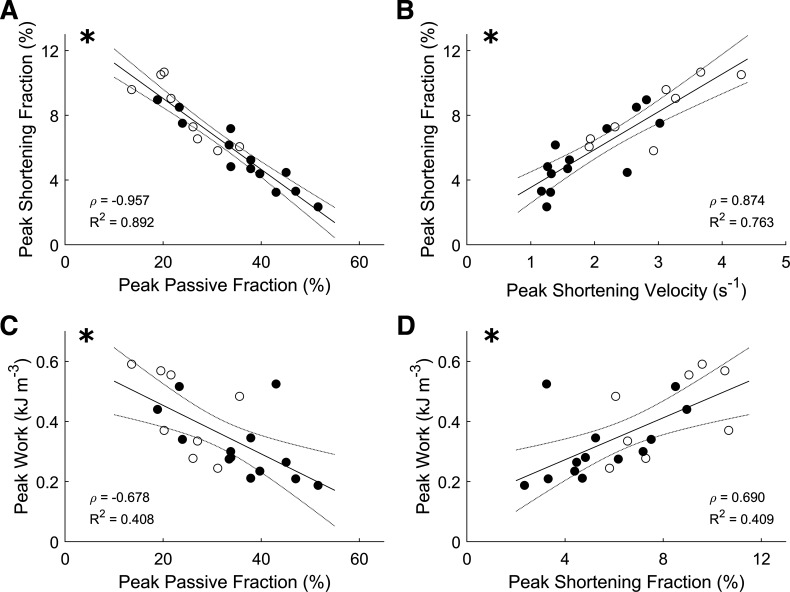
Correlations among mechanical parameters. The plotting convention is the same as in Fig. 2, where open symbols indicate CON group; filled symbols indicate FAIL group, and statistical significance of the correlation is indicated by the presence of an * at the *top left* corner. CON, control; FAIL, failing.

A negative correlation between peak shortening fraction and peak passive fraction was observed, and both parameters correlated with peak work (Fig. 4, *A*, *C*, and *D*). The peak shortening fraction was positively correlated with peak shortening velocity (Fig. 4*B*).

**Figure 5.**
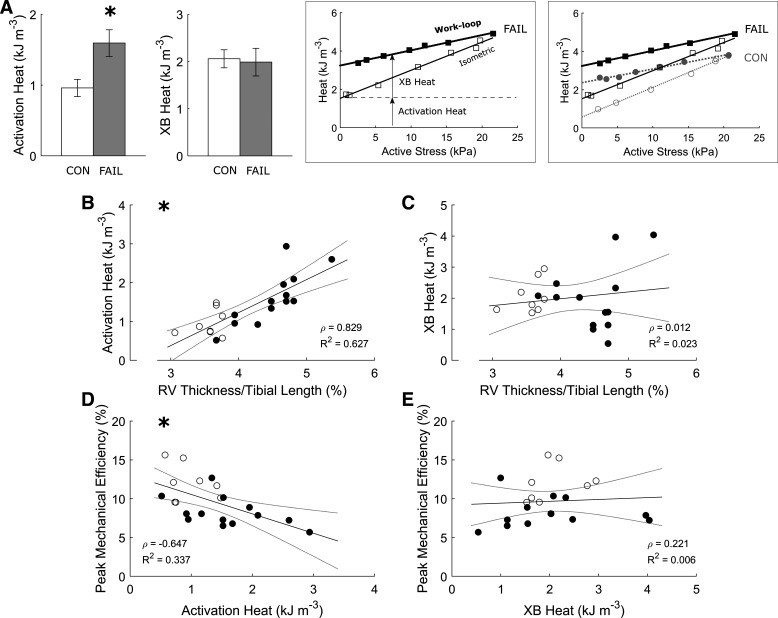
Correlations between heat and mechanical efficiency. *A*: averages of activation heat and cross-bridge (XB) heat are shown for both CON (*n* = 8) and the FAIL (*n* = 13) groups, where *statistically significant difference between groups. The *left* inset shows heat-stress relations from a representative FAIL trabecula for both isometric and work-loop contractions, in which both sets of data were fitted using linear regression, and both activation heat and cross-bridge heat were estimated at the active stress that gave peak mechanical efficiency (as indicated by the arrows). The *right* inset shows heat-stress relations from representative CON and FAIL trabeculae for both isometric and work-loop contractions. For *B–E*, the plotting convention is the same as in Fig. 2, where open symbols indicate CON group; filled symbols indicate FAIL group, and statistical significance of the correlation is indicated by the presence of an * at the *top left* corner. Values are presented as means ± SE. CON, control; FAIL, failing; RV, right ventricle.

Activation heat, but not cross-bridge heat, was greater in the FAIL group (Fig. 5*A*). Activation heat correlated positively with RV thickness (Fig. 5*B*), whereas peak mechanical efficiency varied inversely with activation heat (Fig. 5*D*). Consistent with this collection of results, cross-bridge heat was uncorrelated with normalized RV thickness (Fig. 5*C*), and peak mechanical efficiency was likewise uncorrelated with cross-bridge heat (Fig. 5*E*).

### Cross-Bridge Efficiency and Stiffness

**Figure 6.**
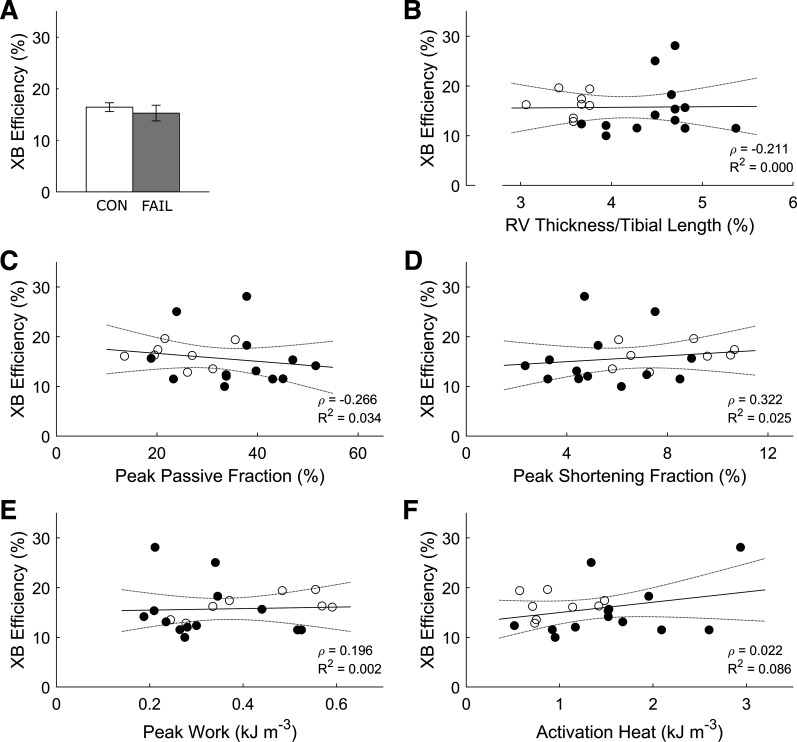
No correlations between cross-bridge efficiency and key indices of energetics. Cross-bridge (XB) efficiency, calculated as the ratio of work to the sum of work and cross-bridge heat (Fig. 5*A* inset), was not statistically different between CON (*n* = 8) and the FAIL (*n* = 13) trabeculae (*A*) and did not correlate with the indices shown in *B–F*. Values are presented as means ± SE. CON, control; FAIL, failing; RV, right ventricle.

**Figure 7.**
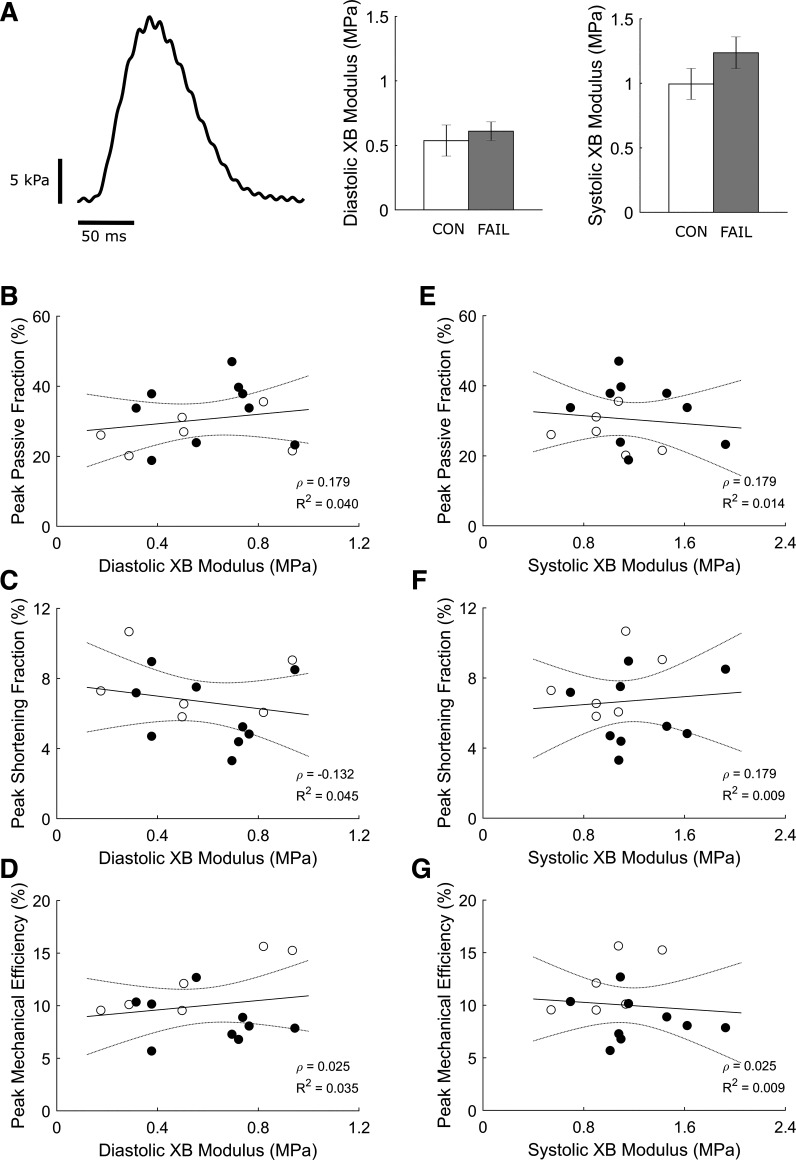
No correlations between cross-bridge dynamic stiffness and indices of mechano-energetics. *A*: single perturbed twitch stress is shown where the amplitude of oscillation provides a measure of cross-bridge dynamic stiffness. Average values of dynamic moduli at diastole and peak systolic stress from CON (*n* = 6) and the FAIL (*n* = 9) trabeculae are shown. *B–G*: show an absence of dependence of key indices of mechano-energetics on dynamic cross-bridge modulus. Values are presented as means ± SE. CON, control; FAIL, failing; XB, cross-bridge.

Cross-bridge efficiency was not different between groups (Fig. 6*A*) and was not correlated with normalized RV thickness (Fig. 6*B*), peak fraction of passive force (Fig. 6*C*), peak shortening fraction (Fig. 6*D*), peak work (Fig. 6*E*), or activation heat (Fig. 6*F*). Dynamic moduli of cross bridges, at diastole and peak systolic stress, were also not different between groups and were not correlated with any measured energetic parameters (Fig. 7).

## DISCUSSION

The present study provides a detailed characterization of cross-bridge mechano-energetics in RV failure by evaluating muscle stress, dynamic stiffness, cross-bridge heat, and cross-bridge efficiency. Experiments were performed on RV trabeculae isolated from MCT-induced pulmonary hypertensive RV failing rats. The trabeculae were subjected to work-loop contractions under physiological conditions using a protocol that allowed partitioning of cross-bridge heat and activation heat, thereby separating cross-bridge efficiency from mechanical efficiency.

### Extension of a Previous Study

To our knowledge, Wong et al. (15) is the only other group that has reported energy efficiency of the same rat model of RV failure. They studied isolated papillary muscles and observed no change in active stress, but a decrease in energy efficiency, in MCT-induced pulmonary hypertensive rats. They showed that the decrease in energy efficiency in muscles of RV failure arises from increased energy consumption and not from cross-bridge mechanics as active stress production, and hence, mechanical work output was not different from the control muscles. They further demonstrated that energy efficiency was negatively correlated with RV wall thickness; that is, the greater the wall thickness indicative of the greater extent of hypertrophy, the lower the energy efficiency. Our findings are consistent with Wong et al. (15). We show that peak active stress is unchanged (Fig. 1*B*) but peak mechanical efficiency is lower (Fig. 1*K*) in RV failing trabeculae. Peak mechanical efficiency is negatively correlated with normalized RV wall thickness (Fig. 2*A*), whereas peak active stress is independent of normalized RV wall thickness (Fig. 2*D*). The negative correlation between peak mechanical efficiency and normalized RV wall thickness is associated with increasing energy output, which we measured from the heat liberation (Fig. 2*B*) and not from cross-bridge mechanics as peak work, which is independent of normalized RV wall thickness (Fig. 2*C*).

Our study extends the findings of Wong et al. (15) on three fronts. First, peak mechanical efficiency is additionally correlated with peak passive fraction, peak shortening fraction, and peak work (Fig. 3), where these mechanical indices are also correlated with one another (Fig. 4). Second, the increased energy consumption in the failing group arises from greater activation heat (Fig. 5*B*), which reflects greater energy expenditure from cellular Ca^2+^ cycling, resulting in lower peak mechanical efficiency (Fig. 5*D*). Thus, these findings provide a conclusion that hypertrophy-associated increase in activation heat is the culprit in RV failure. Last, the decrease in peak mechanical efficiency in the failing group does not imply a diminution of cross-bridge efficiency as shown explicitly in Fig. 6*A* or when indexed as heat (Fig. 5*A*) or cross-bridge modulus (Fig. 7). These findings lead us to conclude that cross-bridge thermodynamics are not affected in pulmonary hypertensive failing RV trabeculae.

### Diastolic Dysfunction in Failing RV Trabeculae

Diastolic dysfunction is a hallmark of RV failing myocardium, characterized by elevated diastolic pressure or passive stress, which is associated with reduced ejection or shortening fraction and slow contractile activity (as detailed in the introduction). Increased passive stress (Fig. 1*C*) has been consistently reported using the same animal disease model (5–7). The mechanism of increased passive stress is attributed to increased fibrotic collagen content in the RV myocardium (4, 8, 28, 29) and reduced phosphorylation of the myofilament protein titin (3, 6).

Increased passive stress in failing RV myocardium is associated with reduced shortening (5, 10, 11). Our results extend literature findings by showing that increased passive stress, as indicated by the passive fraction, in failing RV trabeculae is negatively correlated with peak shortening fraction as well as with peak work output (Fig. 4) and peak mechanical efficiency (Fig. 3*A*). Our finding is supported by two independent studies as follows. Given that the extent of wall tissue shortening is a proxy for stroke volume (30), the lower shortening fraction in failing RV trabeculae is in accord with reduced stroke volume observed in patients with pulmonary arterial hypertension (3, 16). More interestingly, a clinical study that reported reduced efficiency in patients with RV failure has also demonstrated reduced ejection fraction (16).

Slow contractile activity in right-heart failure is an unequivocal finding. We report reduced velocity of shortening and prolonged twitch duration (Fig. 1). The underlying mechanism of the reduced velocity of shortening has been attributed to the shift from the fast (α) to the slow (β) myosin heavy chain isoforms (31–34). This shift of myosin heavy chain isoforms has been reported in the same rat model of RV failure (4, 35–38). Our finding shows that lower velocity of shortening is correlated with the reduction of shortening (Fig. 4*B*) and indirectly correlates with work output (Fig. 4*D*) and reduced mechanical efficiency (Fig. 3*B*). Taken together, we show that diastolic dysfunction and impaired shortening and contractile activity contribute to the reduced mechanical efficiency observed in failing trabeculae.

We noted a previous study (7) which has measured sarcomere length (SL) in isolated myocytes from the MCT-treated rat. They reported a lower diastolic SL in the isolated RV myocytes from the failing group compared with the control group (1.78 µm vs. 1.87 µm). In contrast, from their in vivo hemodynamic measurement, they reported a higher RV diastolic volume (220 µL vs. control 150 µL). These discrepant findings suggest a possibility of the RV diastolic mechanical function to be affected by the different loading conditions. Thus, in our experiments, all trabeculae were required to undergo isometric contractions at the range of muscle lengths (from optimal to minimal lengths) and performed work-loop contractions at a wide range of afterloads at the optimal length. Passive stress was thus determined at the optimal muscle length. In the same study, Fowler et al. (7) attributed decreased creatine kinase expression to diastolic dysfunction and proposed that the mechanism, predominantly through Ca^2+^-independent force production, is via a local reduction in the ATP/ADP ratio. Our study here shows a greater energy expenditure for Ca^2+^-activation of contraction, whereas cross-bridge heat is not different between groups. Thus, our study and Fowler et al. (7) suggest that cardiac energetics for activation processes is a potential target for therapeutic intervention in right-heart failure.

### Preserved Active Stress Production in Failing RV Trabeculae

Despite diastolic dysfunction, as discussed earlier, active force production in failing trabeculae is unaffected. Our results of no difference in the maximal active twitch stress between the control and failing RV trabeculae at physiological temperature (37°C) and the resting heart rate of the rat (5 Hz) are in line with previous studies on isolated RV failing papillary muscles (15) and trabeculae (35), as well as on skinned myocytes from patients with RV failing (17). However, some studies have reported lower active stress in isolated trabeculae under comparable experimental conditions (39) or at sub-physiological temperatures (38, 40–42), whereas another study on skinned cardiomyocytes isolated from patients with pulmonary arterial hypertension showed higher active stress (3). These discrepancies could result from differences in experimental conditions or protocols or the phenomenon that active stress production decreases with muscle cross-sectional area (23, 43, 44). Data interpretation could be confounded if a difference in muscle cross-sectional area between groups exists. Previous studies (15, 35), in line with our current study (Fig. 1*B*), reported no difference in active stress between groups that had no difference in muscle dimensions. The lower active stress reported in a study by Power et al. (39) could be attributed to a 38% higher average cross-sectional area of the failing RV trabeculae (0.062 mm^2^) compared with that of the control trabeculae (0.045 mm^2^). Other studies reporting lower active stress in RV failing muscles (38, 40, 41) did not provide any muscle dimension data between groups.

Despite preserved active stress, many studies have reported impaired Ca^2+^ handling (39–41, 45) and disrupted Ca^2+^-related regulatory protein expressions (t-tubules, junctophilin-2, ryanodine receptor-2; 10, 46) in RV failing myocardium. A possible explanation for preserved active stress is increased Ca^2+^ sensitivity of the myofilament in RV failing myocardium. An increase in myofilament Ca^2+^ sensitivity has been shown in skinned cardiac muscles from the same MCT rat model (35, 45) and skinned cardiomyocytes from patients with RV failure (3).

### Preserved Cross-Bridge Dynamic Stiffness in Failing RV Trabeculae

The magnitude of contractile stress development is dependent on the number of cross-bridge attachments and their cycling kinetics. In the current study, we determined the dynamic modulus of cross bridges throughout the time course of a twitch by measuring dynamic stiffness, which is an index of the number of attached cross bridges (47). We showed no change in dynamic modulus in the failing RV trabeculae (Fig. 7), suggesting preserved status of cross-bridge cycling properties. Our findings on isolated intact trabeculae contracting isometrically are comparable with the results of previous studies using a quick length-change protocol that demonstrated unaffected cross-bridge properties in skinned cardiac strips isolated from hypertrophied RV ferret hearts induced by pulmonary artery banding (48) or in skinned myocytes from patients with RV failing (17). Although those studies were performed on skinned preparations where the cellular membrane structures are completely removed, there is evidence that the cross-bridge kinetics in intact papillary muscles were not altered by the skinning procedure (49), implying compatibility of results between intact and skinned preparations.

We quantified diastolic and systolic dynamic moduli and found no correlations with peak passive fraction, peak shortening fraction, or peak mechanical efficiency (Fig. 7). Despite increased passive fraction and reduced shortening fraction, the number of cycling cross bridges as assessed from the measurement of dynamic stiffness is not different between groups. These results demonstrate that diastolic dysfunction, reported arising from reduced phosphorylation of titin (3, 6) and increased collagen content (4, 8, 28, 29), does not affect cross-bridge dynamic stiffness.

### Reduced Mechanical Efficiency in Failing RV Trabeculae

The active heat liberated by muscle during contraction consists of activation heat and cross-bridge heat. Activation heat reflects the energy cost of cellular Ca^2+^ cycling predominantly by the sarcoplasmic reticulum Ca^2+^-ATPase (SERCA) with some contribution from the sarcolemmal Na^+^-Ca^2+^ exchanger (NCX) coupled to the Na^+^-K^+^ ATPase, and this heat can be measured as the heat-intercept of the isometric heat-stress relation (18) as illustrated in Fig. 5*A.* Decreased SERCA protein (35, 50, 51) and gene (35, 51) expressions as well as a decreased rate of SERCA activity (51) have been reported in RV failing myocardium. In contrast, increased diastolic Ca^2+^ transients and a prolonged rate of relaxation of Ca^2+^ transients have been shown (40). Given that the SERCA transports two Ca^2+^ per ATP hydrolyzed, whereas the sarcolemmal NCX pumps one Ca^2+^ in synchrony with one ATP hydrolyzed by the Na^+^-K^+^ ATPase (52), Ca^2+^ cycling via NCX is more energetically expensive than via SERCA. In addition, NCX protein and gene expressions have been reported to be unchanged in RV failing myocardium (35). Thus, our finding of increased activation heat in RV failing muscles suggests that the increased energy expenditure for Ca^2+^ cycling processes is contributed by impaired Ca^2+^ handling and a putative increased activity of NCX in this disease model.

Our previous work has demonstrated that the reduced mechanical efficiency is attributed to the increased energy cost associated with the cellular cycling of Ca^2+^ required for the activation of contraction (5). This conclusion is extended by our finding here that a negative correlation exists between activation heat and mechanical efficiency. In the current study, we also observed a negative correlation between peak passive fraction and peak mechanical efficiency (Fig. 3*A*); that is, the greater the peak passive fraction, the lower the peak mechanical efficiency. These collective findings allow us to conclude that both mechanics and energetics contribute to the lower mechanical efficiency. That is, in addition to increased activation heat, increased passive fraction also contributes to the lower mechanical efficiency in RV failure by reducing the shortening fraction (Fig. 4*A*).

### Preserved Cross-Bridge Efficiency in Failing RV Trabeculae

Cross-bridge heat can be determined by subtracting activation heat from the measured total active heat and is shown to be unchanged in the failing RV trabeculae (Fig. 5*A*), indicating the same rate of ATP hydrolysis by actomyosin ATPase to detach attached cross bridges. This is not a surprising finding given that the maximal active twitch stress and dynamic stiffness are not different between groups. With our ability to separate activation heat from total active heat, cross-bridge efficiency can be quantified by dividing work output by the sum of work and cross-bridge heat. In contrast to mechanical efficiency, cross-bridge efficiency reflects the energetic performance purely by actomyosin cross bridge. The observation of unchanged cross-bridge efficiency in failing RV trabeculae arises from there being no significant difference in work output and cross-bridge heat in comparison with those of the control trabeculae. Although passive fraction affects mechanical efficiency, it exhibits no correlations with active stress, dynamic modulus, or cross-bridge efficiency, demonstrating that cross-bridge thermodynamics remains unaffected despite the existence of diastolic dysfunction in failing RV trabeculae. Based on our findings of preserved cross-bridge thermodynamics in RV failure, we suggest that future studies should concentrate on investigating the mechanisms for impaired Ca^2+^ cycling leading to increased energy expenditure for activation of contraction.

### Conclusions

By subjecting trabeculae to force-length work-loop contractions under physiological conditions and to different experimental interventions, our data have revealed that reduced mechanical efficiency is correlated with increased activation heat and passive fraction in isolated RV trabeculae from the MCT-induced hypertensive failing rat hearts. In contrast, cross-bridge dynamic stiffness, and thermodynamics as assessed from work and cross-bridge heat output and cross-bridge efficiency, are preserved.

## GRANTS

This work was supported by the Heart Foundation of New Zealand: Research Fellowships (1692 to K.T. and 1869 to T.P.), Emerging Researcher First Grant from the Health Research Council of New Zealand (21/653, to T.P.), Marsden Fast-Start Grants from the Royal Society of New Zealand (1504 to J.-C.H. and 1703 to K.T.), Sir Charles Hercus Health Research Fellowships from the Health Research Council of New Zealand (20/011 to J.-C.H. and 21/116 to K.T.), and a James Cook Research Fellowship from the Royal Society of New Zealand (UOA1902 to A.J.T.).

## DISCLOSURES

No conflicts of interest, financial or otherwise, are declared by the authors.

## AUTHOR CONTRIBUTIONS

T.P., D.S.L., and J.-C.H. conceived and designed research; T.P. performed experiments; T.P. analyzed data; T.P., K.T., A.J.T., D.S.L., and J.-C.H. interpreted results of experiments; J.-C.H. prepared figures; T.P. and J.-C.H. drafted manuscript; T.P., K.T., A.J.T., D.S.L., and J.-C.H. edited and revised manuscript; T.P., K.T., A.J.T., D.S.L., and J.-C.H. approved final version of manuscript.
